# Determinants of *Campylobacter* infection and association with growth and enteric inflammation in children under 2 years of age in low-resource settings

**DOI:** 10.1038/s41598-019-53533-3

**Published:** 2019-11-20

**Authors:** Md Ahshanul Haque, James A. Platts-Mills, Estomih Mduma, Ladaporn Bodhidatta, Pascal Bessong, Sadia Shakoor, Gagandeep Kang, Margaret N. Kosek, Aldo A. M. Lima, Sanjaya K. Shrestha, Md. Ashraful Alam, Alexandre Havt, Amidou Samie, Richard L. Guerrant, Dennis Lang, Mustafa Mahfuz, Zulfiqar A. Bhutta, Eric R. Houpt, Tahmeed Ahmed

**Affiliations:** 1 Nutrition and Clinical Services Division, icddr,b, Dhaka, Bangladesh; 2 Division of Infectious Diseases and International Health, University of Virginia, Charlottesville, USA; 3 Haydom Lutheran Hospital, Haydom, Tanzania; 4 Armed Forces Research Institute of Medical Sciences, Bangkok, Thailand; 5 University of Venda, Thohoyandou, South Africa; 6 Aga Khan University, Karachi, Pakistan; 7 Christian Medical College, Vellore, India; 8 Asociación Benéfica PRISMA, Iquitos, Peru; 9 Clinical Research Unit and Institute of Biomedicine, Federal University of Ceara, Fortaleza, Brazil; 10 Walter Reed/AFRIMS Research Unit Nepal (WARUN), Kathmandu, Nepal; 11 Foundation for the National Institutes of Health, Bethesda, MD USA; 12 Bloomberg School of Public Health, Johns Hopkins University, Baltimore, MD USA

**Keywords:** Biological techniques, Infection

## Abstract

*Campylobacter* species infections have been associated with malnutrition and intestinal inflammation among children in low-resource settings. However, it remains unclear whether that association is specific to *Campylobacter jejuni/coli*. The aim of this study was to assess the association between both all *Campylobacter* species infections and *Campylobacter jejuni/coli* infections on growth and enteric inflammation in children aged 1–24 months. We analyzed data from 1715 children followed from birth until 24 months of age in the MAL-ED birth cohort study, including detection of *Campylobacter* species by enzyme immunoassay and *Campylobacter jejuni/coli* by quantitative PCR in stool samples. Myeloperoxidase (MPO) concentration in stool, used as a quantitative index of enteric inflammation, was measured. The incidence rate per 100 child-months of infections with *Campylobacter jejuni/coli* and *Campylobacter* species during 1–24 month follow up were 17.7 and 29.6 respectively. Female sex of child, shorter duration of exclusive breastfeeding, lower maternal age, mother having less than 3 living children, maternal educational level of <6 years, lack of routine treatment of drinking water, and unimproved sanitation were associated with *Campylobacter jejuni/coli* infection. The cumulative burden of both *Campylobacter jejuni/coli* infections and *Campylobacter* species were associated with poor growth and increased intestinal inflammation.

## Introduction

*Campylobacter* species are curved, gram-negative bacterial enteropathogens with diverse human and animal reservoirs, which have been associated with linear growth shortfalls in children in low-resource settings^1–3^. There are more than 25 species of *Campylobacter*, of which the thermotolerant variants such as *Campylobacter jejuni* and *Campylobacter coli* are thought to most commonly infect humans^4,5^. There are multiple microbiologic approaches for detection of *Campylobacter* species, including bacterial culture, enzyme immunoassay (EIA), and PCR. While both culture and PCR assays can target *Campylobacter jejuni* and *Campylobacter coli*, PCR is substantially more sensitive. The most commonly used EIA tests have been shown to detect a broader range of *Campylobacter* species^6^.

*Campylobacter* infections in young children have been associated with dysentery, diarrhea, and malnutrition^1,5,7^. Environmental enteric dysfunction is a subclinical intestinal disorder which is highly prevalent in low-resource settings and characterized by intestinal inflammation and alteration in gut structure and function^8–10^. Myeloperoxidase (MPO) concentration in the stool can be used as a quantitative index of enteric inflammation^11^ and previous studies suggests MPO as is a simple, noninvasive, and a direct marker of inflammation^1,12–14^. MPO, an enzyme found in granulocytes, is involved with the release of hypochlorous acid. It induces oxidative tissue damage of host tissue following extracellular phagocytic activation at the inflammatory site, resulting in microbial destruction^15,16^. The increase in mucosal MPO levels can be used as a biomarker in human patients with inflammatory bowel disease^17,18^.

The Etiology, Risk Factors, and Interactions of Enteric Infections and Malnutrition and the Consequences for Child Health and Development (MAL-ED) study is a birth cohort performed at 8 sites in South America, sub-Saharan Africa, and Asia^19^. *Campylobacter* species were originally detected by EIA in this study, and a previous analysis showed a strong association between *Campylobacter* species infection and growth^1^. However, the degree to which this association was specific to *Campylobacter jejuni* and *Campylobacter coli*, other *Campylobacter* species, or both remains unclear. Here, we sought to identify risk factors for *Campylobacter jejuni/coli* infection and assess the association with enteric inflammation and linear growth in children and compare these associations with the burden of infestation by *Campylobacter* species by EIA.

## Results

### General characteristics

**Figure 1:**
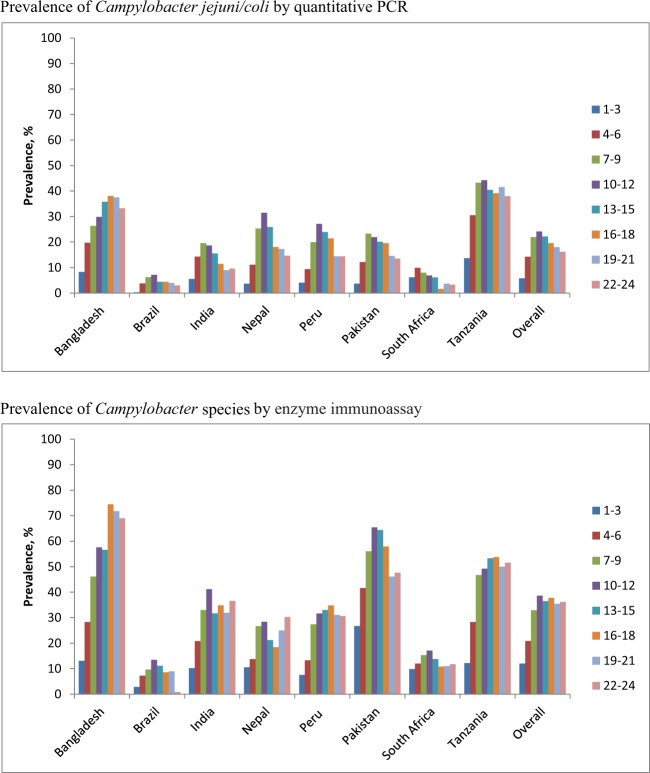
Prevalence of *Campylobacter jejuni/coli* and *Campylobacter* species in stool during 1–24 months by age group.

A total of 1715 participants who completed follow-up to 24 months contributed 34,622 surveillance stool samples tested for *Campylobacter jejuni/coli* by quantitative PCR whereas *Campylobacter* species done by EIA was tested during 1–12, 15, 18, 21, 24 months on 22,614 surveillance stool samples. The demographic characteristics of the study participants are presented in Table 1. The prevalence of *Campylobacter jejuni/coli* and *Campylobacter* species in surveillance stool samples during 1–24 months by sites is shown in Fig. 1. The overall prevalence of *Campylobacter* species infections was approximately twice that of *C. jejuni/coli* infections. Both peaked at approximately one year of age and then subsequently declined for *C. jejuni/coli* and was stable for *Campylobacter* species. The burden was highest in children at the Bangladesh and Tanzania sites.

**Table 1:** General characteristics of the study subjects (n = 1715).

Characteristics, n (%)	Bangladesh	Brazil	India	Nepal	Peru	Pakistan	South Africa	Tanzania	Overall
Male sex	108 (51.4)	89 (53.9)	105 (46.3)	122 (53.7)	105 (54.1)	120 (48.8)	120 (50.6)	105 (50.2)	874 (51.0)
Days of exclusive breastfeeding^†^	143.2 ± 42.7	93.7 ± 57.8	105.4 ± 42.9	92.5 ± 54.5	89.5 ± 61.3	19.9 ± 22.7	38.6 ± 26.3	62.2 ± 35	78.6 ± 57.7
Birth weight (kg)^†^	2.8 ± 0.4	3.4 ± 0.5	2.9 ± 0.4	3 ± 0.4	3.1 ± 0.4	2.7 ± 0.4	3.2 ± 0.5	3.2 ± 0.5	3.0 ± 0.5
Weight for age z-score at Enrollment^†^	−1.3 ± 0.9	−0.2 ± 1	−1.3 ± 1	−0.9 ± 1	−0.6 ± 0.9	−1.4 ± 1	−0.4 ± 1	−0.1 ± 0.9	−0.8 ± 1.1
Length for age z−score at Enrollment^†^	−0.96 ± 1	−0.8 ± 1.1	−1 ± 1.1	−0.7 ± 1	−0.9 ± 1	−1.3 ± 1.1	−0.7 ± 1	−1 ± 1.1	−0.9 ± 1.1
Length for age z-score at 24 month^†^	−2.0 ± 0.9	0 ± 1.1	−1.9 ± 1	−1.3 ± 0.9	−1.9 ± 0.9	N/A	−1.7 ± 1.1	−2.7 ± 1	−1.7 ± 1.2
Maternal age (years)^†^	25.0 ± 5.0	25.4 ± 5.6	23.9 ± 4.2	26.6 ± 3.7	24.8 ± 6.3	28.1 ± 5.9	27 ± 7.2	29.1 ± 6.5	26.3 ± 5.9
Maternal weight (kg)	49.7 ± 8.5	62 ± 11.5	50.3 ± 9.3	56.2 ± 8.3	56.3 ± 9.6	50.7 ± 9.6	68 ± 15.3	55.7 ± 8.8	55.9 ± 12
Maternal height (cm)^†^	149.0 ± 5.0	155.1 ± 6.7	151.1 ± 5.2	149.7 ± 5.3	150.2 ± 5.5	153.4 ± 5.7	158.7 ± 6.6	155.9 ± 5.9	152.9 ± 6.6
Maternal educational level < 6 y	133 (63.3)	22 (13.3)	80 (35.2)	59 (26)	44 (22.7)	202 (82.1)	5 (2.1)	75 (35.9)	620 (36.2)
Mother has less than 3 living children	160 (76.2)	113 (68.5)	157 (69.8)	199 (87.7)	111 (57.2)	105 (42.7)	141 (59.5)	58 (27.8)	1044 (61)
Ownership of chickens/ducks	3 (1.4)	1 (0.6)	14 (6.2)	73 (32.2)	75 (38.7)	144 (62.3)	87 (37.2)	204 (97.6)	601 (35.4)
Ownership of cows/bulls	1 (0.5)	0 (0)	5 (2.2)	3 (1.3)	0 (0)	146 (59.4)	33 (13.9)	157 (75.1)	345 (20.1)
Routine treatment of drinking water	130 (61.9)	10 (6.1)	7 (3.1)	98 (43.2)	32 (16.5)	0 (0)	12 (5.1)	12 (5.7)	301 (17.6)
Improved drinking water source	210 (100)	165 (100)	227 (100)	227 (100)	184 (94.9)	246 (100)	196 (82.7)	89 (42.6)	1544 (90.0)
Improved latrine	210 (100)	165 (100)	121 (53.3)	227 (100)	66 (34)	197 (80.1)	232 (97.9)	19 (9.1)	1237 (72.1)
Improved floor	204 (97.1)	165 (100)	222 (97.8)	109 (48)	69 (35.6)	81 (32.9)	231 (97.5)	13 (6.2)	1094 (63.8)
Monthly income < $150	69 (32.9)	161 (97.6)	19 (8.4)	106 (46.7)	58 (29.9)	115 (46.8)	179 (75.5)	0 (0)	707 (41.2)

^†^Mean ± Standard deviation.

### Incidence and incidence rate of *Campylobacter* infection

The cumulative incidences of *Campylobacter jejuni/coli* and *Campylobacter* species were 86.1% and 90.0% respectively. The incidence rate per 100 child-months of infections with *Campylobacter jejuni/coli* and *Campylobacter* species during 1–24 month follow up were 17.7 (95% CI: 17.0, 18.5) and 29.6 (95% CI: 28.16, 30.3) respectively. The incidence and incidence rate were highest for Bangladesh and Tanzania sites (Table 2). We identified factors associated with *Campylobacter jejuni/coli and Campylobacter* species detection using negative binomial regression in surveillance stool samples across all sites (Table 3). The incidence rate for infection of *Campylobacter jejuni/coli* in female children was 7% higher [IRR: 1.07 (95% CI: 1.07, 1.14); p = 0.048] than in male children. Shorter duration of exclusive breastfeeding [IRR: 0.98 per additional month (95% CI: 0.95, 0.99); p = 0.035], lower maternal age in years [IRR: 0.99 per year (95% CI: 0.97, 0.99); p < 0.001], mother having no less than 3 living children [IRR: 1.15 (95% CI: 1.05, 1.26); p = 0.002], maternal education not greater than or equal to 6 years [IRR: 1.09 (95% CI: 1.01, 1.17); p = 0.021], lack of treatment of drinking water [IRR: 1.26 (95% CI: 1.14, 1.40); p = 0.002], and unimproved sanitation [IRR: 1.11 (95% CI: 1.00, 1.23); p = 0.043] were associated with infection with *Campylobacter jejuni/coli*. Furthermore, female children, shorter duration of exclusive breastfeeding, enrollment weight-for-age z-score (WAZ), lower maternal age, mother having no less than 3 living children, maternal education not greater than or equal to 6 years, no treatment of drinking water, unimproved sanitation, and household ownership of cattle/poultry were also found to be more strongly associated with *Campylobacter* species as detected by EIA compared to *Campylobacter jejuni/coli* as detected by qPCR. The incidence rate ratio for the sites of Brazil (BR), India (IN), Nepal (NP), Peru (PE), Pakistan (PK), and South Africa (SA) were lower compared to the Bangladesh site.

**Table 2:** Incidence rate per 100 Child-months and cumulative incidence of *Campylobacter jejuni/coli and Campylobacter* species infection by site.

Sites	*Campylobacter jejuni/coli* (PCR)		*Campylobacter* species (EIA)	
	Incidence rate per 100 Child-months (95% CI)	Cumulative incidence	Incidence rate per 100 Child-months (95% CI)	Cumulative incidence
Bangladesh	28.3 (26.2, 30.6)	99.5	44.1 (41.6, 46.8)	100.0
Brazil	4.2 (3.4, 5.1)	47.9	8.2 (6.8, 10)	54.6
India	12.9 (11.7, 14.4)	89.0	28.4 (25.9, 31.3)	90.3
Nepal	18.8 (17.4, 20.2)	96.5	21.2 (19.5, 23.1)	93.0
Peru	16.9 (15.5, 18.3)	94.9	23.2 (21, 25.5)	93.3
Pakistan	16.3 (14.9, 17.7)	92.3	49.7 (47, 52.5)	99.2
South Africa	5.7 (5.0, 6.5)	62.5	13.3 (12.1, 14.6)	83.5
Tanzania	36.8 (34.2, 39.6)	100.0	39.8 (37.3, 42.4)	99.0
Overall	17.7 (17.0, 18.5)	86.1	29.2 (28.1, 30.3)	90.2

Incidence rate per was calculated using negative binomial regression where outcome variables were the number of infection of *Campylobacter jejuni/coli and Campylobacter* species infection and offset variables were log of number of follow up.

**Table 3:** Risk factors for *Campylobacter* detection in monthly surveillance stool samples.

Risk Factors by Category	*Campylobacter jejuni/coli* (PCR)		*Campylobacter* species (EIA)	
	IRR (95% CI)	*p*-value	IRR (95% CI)	*p*-value
**Sex of child**				
Male	Reference		Reference	
Female	1.07 (1.00, 1.14)	0.048	1.07 (1.01, 1.13)	0.014
Duration of EBF (month)	0.98 (0.95, 0.99)	0.035	0.97 (0.95, 0.99)	0.004
Enrollment WAZ	1.00 (0.97, 1.04)	0.814	1.03 (1.01, 1.06)	0.015
Maternal age in years	0.99 (0.98, 0.99)	<0.001	0.98 (0.98, 0.99)	<0.001
**Maternal educational level < 6 y**				
No	Reference		Reference	
Yes	1.09 (1.01, 1.17)	0.021	1.12 (1.05, 1.19)	0.001
**Mother has less than 3 living children**				
Yes	Reference		Reference	
No	1.15 (1.05, 1.26)	0.002	1.25 (1.16, 1.34)	<0.001
**Routine treatment of drinking water**				
Yes	Reference		Reference	
No	1.26 (1.14, 1.4)	0 < 0.001	1.39 (1.27, 1.53)	<0.001
**Improved sanitation**				
Yes	Reference		Reference	
No	1.11 (1.00, 1.23)	0.043	1.16 (1.07, 1.26)	<0.001
**Household ownership of cattle/poultry**				
No	Reference		Reference	
Yes	1.07 (0.97, 1.17)	0.167	1.12 (1.04, 1.21)	0.003
alpha (α)	0.13 (0.11, 0.16)	<0.001	0.05 (0.03, 0.08)	<0.001

Model: Negative binomial regression; Dependent variable: Number of infection during follow up (1–24 m); Offset: Log of total number of follow up; alpha (α): dispersion parameter; Adjusted for site and all variables included in multivariable model.

### Association of *Campylobacter* infections with growth and enteric inflammation

The cumulative burden of both *Campylobacter jejuni/coli* infections [−0.18 difference in 24-month length-for-age z-score (LAZ) for children with high vs. low burden of infection (95% CI: −0.30, −0.06), p = 0.004] and *Campylobacter* species [−0.31 difference in 24-month LAZ (95% CI: −0.46, −0.15), p < 0.001] were associated with poor growth, with a stronger association seen for *Campylobacter* species both overall and for the majority of sites (Table 4). Meanwhile, after controlling for infection with enteroaggregative *E. coli* (EAEC), heat-labile enterotoxin-producing *E. coli* (LT-ETEC), heat-stable enterotoxin-producing *E. coli* (ST-ETEC), *Shigella*/enteroinvasive *E. coli* (*Shigella*/EIEC), both *Campylobacter* species and *Campylobacter jejuni/coli* infections were also clearly and consistently associated with increased enteric inflammation as measured by MPO, with a stronger association seen for *Campylobacter jejuni/coli* (Table 5).

**Table 4:** Association of *Campylobacter jejuni/coli* and *Campylobacter* species infection burden on children growth at 24 months.

Sites	*Campylobacter jejuni/coli* (PCR)		*Campylobacter* species (EIA)	
	Coef. (95% CI)	*p*-value	Coef. (95% CI)	*p*-value
Bangladesh	−0.33 (−0.58, −0.09)	0.008	−0.51 (−0.84, −0.18)	0.002
Brazil	−0.39 (−1.27, 0.48)	0.374	−0.25 (−1.07, 0.57)	0.543
India	−0.28 (−0.61, 0.05)	0.096	−0.17 (−0.49, 0.14)	0.284
Nepal	−0.30 (−0.59, −0.01)	0.042	−0.40 (−0.83, 0.02)	0.062
Peru	−0.07 (−0.40, 0.27)	0.692	−0.40 (−0.77, −0.03)	0.035
South Africa	−0.62 (−1.20, −0.05)	0.034	−0.13 (−0.85, 0.59)	0.719
Tanzania	0.13 (−0.10, 0.36)	0.276	−0.24 (−0.58, 0.11)	0.180
Overall	−0.18 (−0.30, −0.06)	0.004	−0.31 (−0.46, −0.15)	<0.001

Adjusted in linear regression model for sex, WAMI Index (water/sanitation, assets, maternal education, and income); enrollment length-for-age z score; maternal height; poultry/cattle in house, mother has less than 3 living children and site for overall estimate; Dependent variable: length-for-age z score at 24 m*;* Independent variables: *Campylobacter* burden.

**Table 5:** Association between *Campylobacter jejuni/coli* and enteric inflammation (stool myeloperoxidase).

Sites	Stool myeloperoxidase (MPO) concentration			
	*Campylobacter jejuni/coli* (PCR)		*Campylobacter* species (EIA)	
	Coef. (95% CI)	*p*-value	Coef. (95% CI)	*p*-value
Bangladesh	0.20 (0.08, 0.31)	0.001	0.23 (0.13, 0.33)	<0.001
Brazil	0.54 (0.19, 0.90)	0.003	0.49 (0.24, 0.74)	<0.001
India	0.40 (0.27, 0.53)	<0.001	0.24 (0.14, 0.33)	<0.001
Nepal	0.34 (0.23, 0.45)	<0.001	0.24 (0.13, 0.34)	<0.001
Peru	0.26 (0.12, 0.41)	<0.001	0.22 (0.09, 0.35)	0.001
Pakistan	0.28 (0.15, 0.41)	<0.001	0.20 (0.10, 0.30)	<0.001
South Africa	0.37 (0.19, 0.55)	<0.001	0.17 (0.05, 0.30)	0.007
Tanzania	0.24 (0.13, 0.34)	<0.001	0.09 (−0.01, 0.19)	0.088
Overall	0.29 (0.24, 0.34)	<0.001	0.20 (0.16, 0.24)	<0.001

Adjusted in the in GEE model for sex, age, WAMI Index (water/sanitation, assets, maternal education, and income); enrollment length-for-age z score; maternal height; number of children, poultry/cattle in house, seasonality, site for overall estimate, some alternative pathogens (EAEC, LT-ETEC, ST-ETEC, Shigella/EIEC), and age as time variable. Dependent variable was log(MPO); Independent variables: presence of *Campylobacter* at each months.

## Discussion

In this prospective multisite birth cohort study, we documented a high burden of *Campylobacter* infections, with most of the children having *Campylobacter* detected in a monthly surveillance stool sample by one year of age at seven of the eight sites. The burden was highest in Bangladesh and Tanzania and consistent with prior studies^20,21^. Our study also shows the incidence rates are more among Bangladesh and Tanzania than other sites (for instance, Brazil, India, Nepal, Peru, Pakistan, South Africa). Overall, the incidence of *Campylobacter* species infections is approximately 65% higher than that of *Campylobacter jejuni/coli* alone. In keeping with data from previous study by Amour *et al*., showed that promotion of exclusive breastfeeding, drinking water treatment, improved latrines, and targeted antibiotic treatment may reduce the burden of *Campylobacter* species infection [1], our study found that *Campylobacter* infections were significantly associated with female sex, shorter duration of exclusive breastfeeding, lower maternal age, less maternal education, lack of treatment of drinking water, and unimproved sanitation. Birth weight-for-age is a marginal predictor for *Campylobacter* species whereas the presence of *Campylobacter* species is associated with growth shortfalls^1^. Among malnourished children from a case control study where the cases comprised of children with weight-for-age z score (WAZ) < −2 aged 6–23 months in Dhaka, prevalence of *Campylobacter* was high compared to healthy (control) children [weight-for-age z score (WAZ) > −1] but the adjusted effect size was not statistically significant^20^.

The association of *Campylobacter jejuni/coli* infection with nutritional status and fecal MPO concentrations of children less than 2, after controlling for seasonality and potential confounders including socio-economic and demographic factors, suggests that *Campylobacter jejuni/coli* have influence on childhood malnutrition and intestinal inflammation^20^. This finding suggests that *Campylobacter* can drive intestinal inflammation, which is partly due to altering of the composition of the intestinal microbiota, impairing the intestinal barrier, and priming the intestine for chronic inflammatory responses and is consistent with results of other studies^1,7^. Meanwhile, the association with growth was stronger for *Campylobacter* species than with *Campylobacter jejuni/coli*, which might suggest that non-*jejuni/coli* species are more strongly associated with poor growth. However, the association with inflammation is stronger for *Campylobacter jejuni/coli*. Further elucidation of the prevalence, clinical relevance, and mechanisms for association with poor growth are needed for diverse *Campylobacter* species.

There were some limitations in this paper. As an observational cohort study, the causality of the associations between *Campylobacter* infections and both intestinal inflammation and linear growth cannot be confirmed but can be inferred based on a number of criteria, including the appropriate adjustment of the models for potential confounders, the strength and consistency of the associations, and the biological plausibility. We have not established a temporal relationship between infections and the outcomes, which would require structured longitudinal models, however previous analyses using such approaches found consistent results for the association between *Campylobacter* infections and linear growth^2^. These findings suggest that *Campylobacter* species other than *Campylobacter jejuni/coli* may be more strongly associated with child growth shortfalls. Further work is needed to directly assess the epidemiology and impact of individual *Campylobacter* species. Secondly, interventions that reduce exposure to these diverse *Campylobacter* species need to be identified, and the impact of these interventions on child growth need to be assessed. We compared the associations of *Campylobacter* species and *Campylobacter jejuni/coli* infections with growth and found that other non-*jejuni/coli* species were also associated with poor growth, however, the absence of direct microbiologic assays for specific non-*jejuni/coli Campylobacter* species limited our ability to understand what species or group of species are driving these associations.

In conclusion, children harboring risk factors such as female sex, shorter duration of exclusive breastfeeding, lower maternal age in years, maternal education not greater than or equal to 6 years, mother having less than 3 living children, lack of routine treatment of drinking water, unimproved sanitation, and ownership of cattle/poultry were more prone to *Campylobacter* infection and thereby have compromised nutritional status; such infection was higher in Bangladesh and Tanzania compared to other sites. The burden of *Campylobacter* was associated with increased enteric inflammation among children in the first 2 years of life. *Campylobacter* species had a stronger association with growth whereas the association with inflammation was strongest for *Campylobacter jejuni/coli*.

## Method

### Study design and participants

The MAL-ED study design and methodology have been previously described^19^. Briefly, children were enrolled November, 2009 to February, 2012 from the community within 17 days of birth at eight locations: Dhaka, Bangladesh; Vellore, India; Bhaktapur, Nepal; Naushero Feroze, Pakistan; Venda, South Africa; Haydom, Tanzania; Fortaleza, Brazil; and Loreto, Peru. Children were included if the maternal age was 16 years or older, their family intended to remain in the study area for at least 6 months from enrolment, they were from a singleton pregnancy, and they had no other siblings enrolled in the study. Children with a birthweight or enrolment weight of less than 1500 gm and children diagnosed with congenital disease or severe neonatal disease were excluded. The study was approved by the Research Review Committee and the Ethical Review Committee of icddr,b (Bangladesh), the Local Institutional Review Board at the Federal Universisty of Ceará and the national IRB Conselho Nacional de Ética em Pesquisa (Brazil), the Christian Medical College Institutional Review Board and the Emory University Institutional Review Board (India), the Nepal Health Research Council and Walter Reed Institute of Research (Nepal), the Ethics Committee of Asociacion Benefica PRISMA, the Regional Health Directorate of Loreto and the IRB of Johns Hopkins Bloomberg School of Public Health (Peru), the Ethical Review Committee of Aga Khan University (Pakistan), the Institutional Review Boards at the University of Venda (South Africa), the National Institute for Medical Research (Tanzania), and the Institutional Review Board of the University of Virginia (UAS). Written informed consent was obtained from the parents or legal guardian of every child^19,22^. All methods were performed in accordance with the relevant guidelines and regulations.

### Data collection

Household demographics, presence of siblings, maternal characteristics, and other data on the child’s birth and anthropometry were obtained at enrollment^19^. The socioeconomic status (SES) of families was assessed at 6, 12, 18, and 24 months. SES score, the water/sanitation, assets, maternal education and income (WAMI) index was developed using composite indicators including the variables such as access to improved water and sanitation, eight selected assets, maternal education, and household income^23^. Improved water and sanitation were defined following World Health Organization guidelines^24^. Treatment of drinking water was defined as filtering, boiling, or adding bleach^1^. Anthropometric measurements and vaccination history were collected monthly. Details of illness and child feeding practices were collected during twice-weekly household visits^25^. Stool samples were collected monthly and were preserved, transported, and processed at all sites using harmonized protocols^26^. Child anthropometry was measured using standard scales (seca gmbh & co. kg., Hamburg, Germany). Length-for-age Z score (LAZ) was calculated through the use of the 2006 WHO standards for children^27^. The Z-score scale, calculated as (observed value - average value of the reference population)/standard deviation value of reference population, is linear and therefore a fixed interval of Z-scores has a fixed length difference in cm for all children of the same age. Z-scores are also sex-independent, thus permitting the evaluation of children’s growth status by combining sex and age groups^28^.

### Laboratory testing

Stool samples were collected without fixative by field workers and raw stool aliquots were kept at −80 °C before nucleic acid extraction. All lab testing was done at the site specific laboratories^11,14^. Stool samples were assayed for *Campylobacter* species by enzyme immunoassay (ProSpecT, Remel, Lenexa, KS, USA). In addition, myeloperoxidase (MPO) (Alpco, Salem, New Hampshire) was measured using commercially-available Enzyme Linked Immunosorbent Assay (ELISA) kits following the instructions of the manufacturers^1,8^. *Campylobacter jejuni/coli* were detected in the stool samples by quantitative PCR targeting the *cadF* gene using the TaqMan Array Card (TAC) platform, a compartmentalized probe-based real-time PCR assays for detecting enteropathogens in fecal samples, as previously described^22,29^. The analytic cutoff of each pathogen was a quantification cycle (Cq) of 35; thus, a Cq < 35 was considered positive^20,30^.

### Statistical methods

All statistical tests were performed in STATA 14 (Stata Corporation, College Station, TX). *Campylobacter* burden was defined as the number of pathogens detected divided by the number of stools collected and was scaled divided by (10^th^ vs 90^th^ percentile). Descriptive statistics such as proportion, mean and standard deviation for symmetric data, and median with inter‐quartile range (IQR) for asymmetric quantitative variables were used to summarize the data. Chi-square and proportion test was used to see the association between two categorical variables and t-test was used to see the mean difference between two groups for symmetric distribution. Cumulative incidence of *Campylobacter jejuni/coli* and *Campylobacter* species was defined as the proportion of subjects who were infected at least once during the study period. Incidence rates and risk factors associated with *Campylobacter* detection in surveillance stool samples were calculated using negative binomial regression models due to over dispersion. In the final multiple negative binomial regression model, the following variables were considered for inclusion using stepwise forward selection: child sex, duration of exclusive breastfeeding in months, enrollment weight for age z-score, maternal age in years, maternal education greater than or equal to 6 years, mother having less than 3 living children, routine treatment of drinking water, improved sanitation, and household ownership of cattle/poultry. The MPO values were log‐transformed before the analysis. We excluded children from the Pakistan site for growth analysis, owing to bias noted in a subset of length measurements at this site. Seasonality was calculated via the terms *sin*(2mπ/12) + *cos*(2mπ/12), where “m” is the calendar month^1,31^. Associations between *Campylobacter* infection and inflammation was estimated using generalized estimating equations to fit regression models after adjusting for seasonality, sex, age, water/sanitation, assets, maternal education, and income (WAMI) index; enrollment length-for-age; maternal height; poultry/cattle in house, some alternative pathogens which were significantly associated with log(MPO) such as enteroaggregative *E. coli* (EAEC), heat-labile enterotoxin-producing *E. coli* (LT-ETEC), heat-stable enterotoxin-producing *E. coli* (ST-ETEC), Shigella/enteroinvasive *E. coli* (*Shigella*/EIEC), and site for overall estimate and age in month as time variable^32^. The Gaussian family with identity link was used for the continuous outcome of log(MPO). To access and compare the associations of *Campylobacter jejuni/coli* and *Campylobacter* species infection burden on growth at 24 months of age, we used multi-variable linear regression after adjusting for site and the necessary covariates.
